# Autologous Advanced Tenon Grafting Combined with Conjunctival Flap in Scleromalacia after Pterygium Excision

**DOI:** 10.1155/2015/547276

**Published:** 2015-04-23

**Authors:** Jong Soo Lee, Min Kyu Shin, Jong Ho Park, Young Min Park, Margaret Song

**Affiliations:** ^1^Department of Ophthalmology, School of Medicine, Pusan National University and Medical Research Institute of Pusan National University Hospital, Busan 602-739, Republic of Korea; ^2^Department of Ophthalmology, Yangsan Pusan National University Hospital, Yangsan 626-870, Republic of Korea; ^3^Department of Dermatology, School of Medicine, Pusan National University and Medical Research Institute of Pusan National University Hospital, Busan 602-739, Republic of Korea

## Abstract

*Purpose*. To evaluate the efficacy of autologous tenon grafting combined with conjunctival flap as a treatment for scleromalacia or scleral thinning after pterygium excision without any additional donor graft tissue.* Methods*. Twenty-six cases underwent autologous advanced tenon grafting combined with sliding or rotating conjunctival flap for scleromalacia after pterygium surgery ranging from 2 years to 30 years. The extent of scleral defect measured from 2.0 mm to 6.8 mm in diameter. The cosmetic outcome was defined as complete resolution of scleromalacia or completely conjunctival reepithelialization and firm adhesion between subtenon and scleral tissue over scleral thinning without significant complications.* Results*. All cases achieved the covering of conjunctival and tenon or subtenon tissue over scleromalacia or scleral thinning with this procedure. Preoperative pain, inflammation, and choroidal exposure disappeared after surgery. Immediate postoperative complications, such as large wound dehiscence or reopening of the scleral wound, did not occur in any of the patients. There were no significant clinical complications during the mean postoperative follow-up period of 14.17 months in all cases.* Conclusions*. We obtained excellent outcome with fewer complications after autologous advanced tenon graft and conjunctival flap, without an additional donor graft, in scleromalacia or scleral thinning caused by previous pterygium excision.

## 1. Introduction

Scleromalacia commonly occurs after pterygium excision [1] and results in staphyloma formation, scleral perforation, and uveal exposure. Recently, there is an increasing tendency of scleral ischemic changes after pterygium surgery related to using mitomycin C for reducing the risk of recurrence [2–4]. When the choroid is exposed, any reinforced operative treatment for the scleral thinning or scleromalacia is required due to a high risk of secondary infection and possible prolapse of ocular contents, even with minor trauma. Thus, many operative methods and grafting materials have been introduced for the treatment of these defects.

The sclera [5], temporalis fascia and muscle [6], periosteum [7], and dermis [8] have been previously used as grafting materials for scleral defects. However, selecting the donor graft material is not only limited with respect to obtaining the perfect graft material, but it is also sometimes difficult to obtain these various donor graft materials at the time that the scleral reinforcement procedure is performed. For instance, although the scleral donor tissue is strong, is flexible, is easy to handle, and can be preserved for several months, the lack of adequate vascularization in sclera may result in necrosis and sloughing and finally lead to failure of scleral grafts [9].

There is a clinical need for new graft materials that compensate for the disadvantages and limitations of conventional donor materials. Thus, we utilized an autologous advanced tenon graft as a strategy for maintaining the integrity of the globe in patients with scleral thinning or scleromalacia after pterygium excision without any donor graft materials. Herein, we report our experiences of autologous tenon graft with conjunctival flap, with more than 12 months of follow-up.

## 2. Materials and Methods

### 2.1. Patients

We retrospectively reviewed the medical records at Pusan National University Hospital, Busan, Republic of Korea, between April 2009 and November 2012. Twenty-six eyes of 26 consecutive patients with scleromalacia or scleral thinning after pterygium excision were included. All patients presented with clinical signs of noninfectious scleromalacia or scleral thinning with impending globe rupture. The major indication for surgical intervention was severe scleral thinning, uveal exposure, and impending globe perforation. Patients with previous strabismus surgery, filtering operation, or any other procedure that could result in poor tenon tissue harvesting were excluded from the study. The limitation of indication of this procedure was any scleromalacia with acute or chronic inflammatory reaction, or marked deficiency of adjacent conjunctival tissues. Microbiologic analysis for isolation of the original organism of scleritis was performed with scleral debridement materials.

The clinical outcome was assessed using anterior segment examination and best-corrected visual acuity (BCVA), and surgical complications were recorded. Each patient or their legal representative received a comprehensive explanation about the surgery and its implications, and written informed consent was obtained prior to the surgery. This study was approved by Institutional Review Board Ethics Committee (E-2013048) in the Pusan National University Hospital. This investigation adhered to the tenets of the Declaration of Helsinki.

### 2.2. Data Recorded Postoperatively

Each patient received a comprehensive ophthalmic examination including measurement of distant BCVA using Snellen charts at 4 m and anterior segment slit-lamp examination. Characteristics of preoperative ocular symptoms were recorded in the chart.

### 2.3. Surgical Technique

All operations were performed by one surgeon (Jong Soo Lee) under subconjunctival and topical anesthesia. The main surgical technique consists of sliding or rotating conjunctival flap and making advanced tenon flap from healthy conjunctival and tenon tissue nearby scleromalacia lesion.

First, we surgically removed all melting scleral tissues, calcific deposits, and the involved conjunctival tissues in and around the affected sclera. After complete removal of necrotic tissue, the size of the scleral thinning or scleromalacia was measured with calipers. The healthy adjacent tenon tissue was undermined and trimmed to fit the scleral defect. The advanced graft of tenon tissue was usually obtained from the superonasal or inferonasal site, rather than the medial site, because the medial tenon tissue is less movable than the mentioned two sites of tenon tissues. The surgeons were also aware that tightening of the advanced tenon flap may limit ocular movement. If we use the medial tenon tissue to cover the scleral thinning, this advanced medial tenon flap may often induce the limitations of eye movement, especially in lateral gaze. So, we have to reduce the tensile strength by medial advanced tenon tissue, for example, making long and thin thickness of advanced tenon graft.

**Figure 1:**
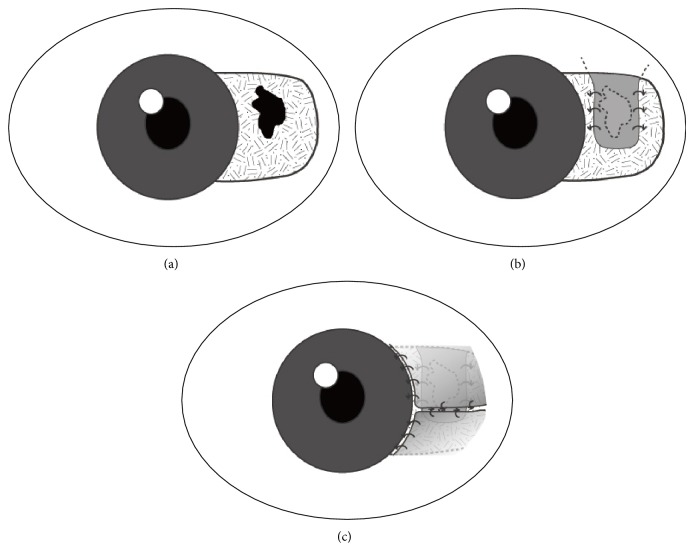
Diagram of the surgical procedure. (a) Area of scleral thinning. (b) Advanced tenon flap harvested from the superonasal site of the scleral thinning and complete covering of the scleromalacia boundary. (c) Sliding conjunctival flap was made over the area of the tenon graft with fixation sutures between conjunctiva and sclera.

The devitalized or thinning scleral defect was covered by the advanced flap of tenon graft and fixed to the margin of bare scleral lesion with Vicryl 8-0 interrupted sutures (Johnson and Johnson, New Brunswick, NJ). Thus, it was important to make a fixed suture of advanced tenon graft on the healthy margin of the scleromalacia against the original tension of tenon to prevent slipping of the advanced tenon flap. Afterwards, the scleromalacia was covered completely by the adjacent healthy upper or lower nasal advanced tenon flap, and then rotating or sliding bulbar conjunctival flap was made from healthy adjacent conjunctiva and fixed with Prolene 9-0 interrupted sutures (Johnson and Johnson, New Brunswick, NJ) to prevent wound dehiscence between the conjunctival and scleral tissue by ocular movement (Figure 1).

After all the procedures were completed, dexamethasone 0.1% eye drop (Maxidex; Alcon Laboratories, Inc., Fort Worth, TX) and ofloxacin 0.3% eye drop (Tarivid; Santen Co., Ltd., Osaka, Japan) were applied on the eye with bandage. The bandage was opened the next day and topical fluorometholone 0.1% (Flumetholon; Santen Co., Ltd., Osaka, Japan), moxifloxacin 0.5% (Vigamox; Alcon Laboratories, Inc., Fort Worth, TX), and carboxymethylcellulose 0.5% (Refresh Plus; Allergan, Inc., Irvine, CA) were prescribed 4 times daily starting 1 day after the surgery in all eyes and gradually tapered over a 1-month period according to graft site status. Conjunctival stitches were usually removed 2~5 weeks (average, 2.5 weeks) after surgery.

### 2.4. Postoperative Visits

After the operation, subjective symptoms of patient and surgical wound status were recorded at postoperative days 1 and 7 and at 1, 3, and 6 months or final visit when clinically indicated.

## 3. Results

In all, 26 eyes of 26 patients (7 men [27%] and 19 women [73%]; age range, 46–79 years) underwent autologous tenon graft and conjunctival flap surgery. All patients had a common etiology of scleromalacia in that patients had previous history of pterygium surgery over a wide interval of 2 years to 30 years (mean, 10.3 ± 6.7 years). The extent of scleral defect measured from 2.0 mm to 6.8 mm in diameter. The mean postoperative follow-up period was 14.7 months (±7.83), ranging from 6 to 38 months. The mean preoperative visual acuity was 0.26 ± 0.23 by log MAR; and the visual acuity was slightly improved to 0.25 ± 0.38 after surgery. The change of visual acuity between pre- and postoperative ocular surface is not significant.

There was no evidence of bacterial or fungal infection in scleromalacia or scleral thinning by culture. Unacceptable cosmetic appearance due to severe choroidal exposure and scleral thinning was present in eight patients. Other main preoperative symptoms included foreign body sensation, ocular pain with calcified deposit, and impending scleral perforation. The size of the diameter of scleral lesion varied (Table 1). The cosmetic outcome was defined as complete resolution of scleromalacia or completely conjunctival reepithelialization and firm adhesion between subtenon and scleral tissue over scleral thinning.

There were no significant ocular complications, such as penetrating injury or globe rupture and secondary infection during or after this operation. Complete reepithelialization of ocular conjunctival surface was obtained within 4 weeks after surgery.

**Figure 2:**
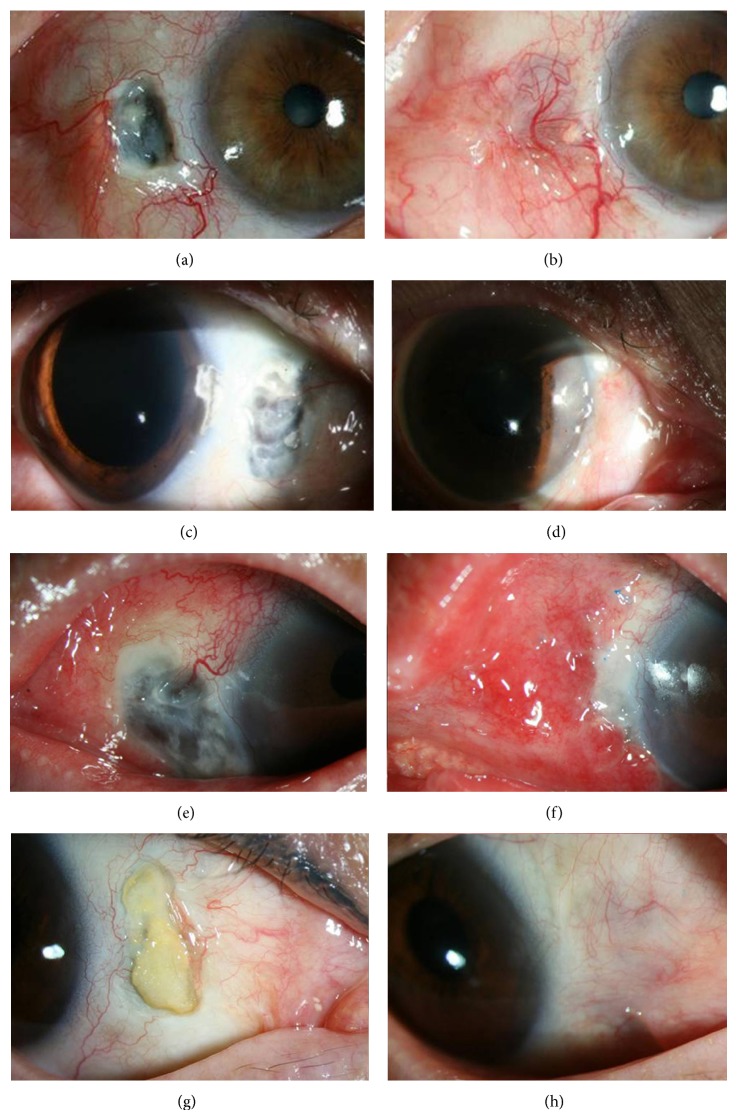
Case 1: (a) preoperative photograph, a 2.9 × 5.3 mm sized scleral thinning with visible uvea. (b) Successful reconstruction of ocular surface at 2 months after surgery. Case 2: (c) preoperative photograph, a 3.8 × 6.3 mm sized scleral melt with visible choroid. (d) Postoperative successful reconstruction of ocular integrity at 10 months after surgery. Case 3: (e) preoperative photograph, a 4.8 × 6.8 mm sized scleral thinning with uveal exposure. (f) Successful tenon tissue graft and conjunctival flap without complications at postoperative 4 weeks. Case 4: (g) preoperative photograph, a 2.1 × 5.5 mm sized scleral thinning with large calcified plaque. (h) Successful reconstruction of ocular surface at 12 months after tenon graft combined with conjunctival flap.

Immediately after the surgery, the most common subjective symptoms were pain and redness. Ocular discomfort including pain usually resolved within 1~2 months in all cases, and sometimes redness lasted as long as 4 months. Transient diplopia appeared in three cases but spontaneously disappeared after 1 week. Significant complications such as wound dehiscence, detachment of tenon graft tissue, immune reaction, or cosmetic problems did not occur. Tectonic success of the graft was achieved in all patients during the follow-up period (Figure 2). Preoperative subjective symptoms were relieved and patients were satisfied with the postoperative functional and cosmetic results at 12 months or greater after the surgery.

## 4. Discussion

Over the last decade, numerous donor tissues and synthetic materials have been used to reconstruct the ocular surface [10–13]. However, no material has been found to be universally acceptable to date. The sclera has a natural curvature allowing it to neatly blend with host scleral tissue. Hence, the sclera was used as graft material in cases with scleromalacia, impending rupture of the eye, or traumatic scleral dehiscence [9]. The advantage of a scleral graft is that it has avascular structure and is well tolerated with little inflammatory reaction. However, the lack of vascularization may result in graft failure, and often a large graft size is difficult to obtain.

Scleral thinning, caused by previous pterygium surgery, showed favorable results after tectonic corneal lamellar grafting [14]. The tectonic capabilities of corneal tissue reduce the risk of remelting and offer a higher resistance to infection than other graft materials. However, cornea has a higher radius of curvature than any other donor tissue, and placement of a corneal patch in the scleral bed usually results in a more elevated lesion. Additionally, the transparent nature of a corneal patch may result in a less cosmetically acceptable result.

Amniotic membrane consists of a thick basement membrane and is endowed with anti-inflammatory [15], antifibrotic [16], and epithelialization-promoting properties. However, only this membrane may not provide adequate tectonic rigidity and is susceptible to rapid loss of donor tissue.

Fascia lata can be prepared in any size or any shape and is suitable for scleral patching. It provides good tectonic support and cosmetic success and may be used as either an autograft or homograft. However, fascia lata has an increased risk of necrosis because of its poor vascularization and requires an additional surgery to obtain the graft tissue.

Split thickness of dermal graft has the ability to survive on an avascular surface. Although dermal grafts are also flexible and nonbulky with good tensile strength, they have the disadvantage of being cosmetically unacceptable and are unsuitable for use in infective cases [9]. Also, dermal grafts require an additional surgery and induce a higher risk morbidity.

Therefore, we planned and attempted to use autologous advanced tenon tissue near the scleromalacia or scleral thinning without any secondary additional donor graft tissue. Tenon tissue as graft materials can be easily obtained from the patient without the need of additional donor site preparation. We made an advanced tenon flap, which has abundant blood supply and causes very little immune reaction. As the advanced tenon flap has a better survival rate of graft material due to abundant vasculature of flap, the firm adhesion of tissues between sclera and conjunctival surface was made simulated with lack of tensile strength of advanced tenon flap tissue. Thus, we think that the morphological outcome of this procedure is better and safer than that of conjunctival flap alone. Another distinguishing advantage of tenon tissue over the scleral graft is that relatively large defects can also be indicated. In our study, the largest diameter of scleral defect was measured to be more than 6 mm in 8 patients (30.8%). Despite these large defects, no patients suffered from graft necrosis or wound dehiscence. No wound dehiscence occurs due to plentiful blood supply from autologous tenon and subtenon tissue, resulting in restoration of the ocular surface and maintenance of the ocular tectonic support. According to our result, none of the patients showed wound dehiscence or granuloma formation. We would like to recommend to the beginner surgeon that this technique might be useful to patients with small to moderate sized scleral thinning rather than those with large sized over 10 mm impending perforations or full thickness perforations.

Additionally, we used a sliding or rotating conjunctival flap over the advanced tenon graft in attempt to provide epithelial coverage and to minimize the risk of postoperative graft failure. The reformation of the ocular conjunctival surface prevents conjunctival epithelial breakdown and subsequent melt and infection. Full reconstruction of ocular integrity and the conjunctival surface also results in excellent cosmetic outcome.

On the basis of our clinical experiences, autologous advanced tenon graft combined with conjunctival flap would be beneficial for the treatment of scleromalacia or scleral thinning without inflammation.

## Figures and Tables

**Table 1:** The main preoperative symptoms included the size of scleral thinning in 26 patients.

Symptoms	Cases	Range of scleral lesion (mm) (width-length)
Cosmetic problem (choroidal exposure + scleral thinning)	8	2.8–6.7
Foreign body sensation (calcified plaque + scleral thinning)	7	2.0–6.5
Ocular pain with calcified deposit	7	2.1–5.5
Impending scleral perforation	4	4.8–6.8
